# Evaluating the Differential Risk of Contrast-Induced Nephropathy Among Diabetic and Non-diabetic Patients Following Percutaneous Coronary Intervention

**DOI:** 10.7759/cureus.53493

**Published:** 2024-02-03

**Authors:** Fahad R Khan, Tariq Nawaz, Wasim Sajjad, Sadam Hussain, Muhammad Amin, Hassan Ali

**Affiliations:** 1 Cardiology, Lady Reading Hospital, Peshawar, Peshawar, PAK; 2 Cardiology, Lady reading Hospital, Peshawar, Peshawar, PAK

**Keywords:** interventional cardiology, coronary artery disease (cad), diabetic patients, primary percutaneous coronary intervention (pci), contrast-induced nephropathy (cin)

## Abstract

Background

Contrast-induced nephropathy (CIN) significantly complicates percutaneous coronary intervention (PCI), with a higher prevalence in diabetic patients. This study compares the incidence of CIN in diabetic and non-diabetic patients undergoing PCI.

Material and methods

Conducted at Lady Reading Hospital, Peshawar, PAK, from January to December 2023, this observational study involved 450 adult patients with coronary artery disease (CAD) undergoing PCI. The cohort was categorized based on diabetes status, excluding patients with chronic kidney disease and those on renal replacement therapy. Baseline characteristics documented included age, gender, blood pressure, creatinine levels, and the presence of acute coronary syndrome (ACS). CIN was defined as a ≥25% increase in serum creatinine from baseline within 48-72 hours post-PCI. Data analysis was performed using the Statistical Package for the Social Sciences (IBM SPSS Statistics for Windows, IBM Corp., Version 25.0, Armonk, NY), incorporating descriptive statistics, Chi-square tests, and independent t-tests, with a significance level of p<0.05.

Results

The median age of the study population was 55 years. The cohort comprised 52% male (n=234) and 48% female (n=216). Notably, 33% (n=149) had ACS. Diabetic patients exhibited a significantly higher incidence of CIN post-PCI compared to non-diabetics. The highest incidence of CIN (17%, n=77) occurred in the 70+ age group. The findings highlight the criticality of renal function monitoring and procedural adjustments for diabetic patients.

Conclusion

Diabetic patients demonstrate an increased risk of CIN following PCI. This necessitates the development of tailored prevention strategies for this high-risk subgroup.

## Introduction

The risk of contrast-induced nephropathy (CIN) during percutaneous coronary intervention (PCI) is difficult yet significant having an important impact on both patient outcomes and healthcare in general. CIN, for instance, varies greatly, and those with diabetes mellitus (DM) are significantly more prone to suffer from it [1,2]. Mehran et al. [3] and Stone GW et al. [4] conducted pivotal research that provided light on general risk factors and approaches to prevent CIN. However, the evident risk difference between diabetes and non-diabetic individuals requires additional investigation.

At the crux of this issue is an elusive understanding of diabetes' role in CIN development post-PCI. This gap persists despite the acknowledged increased susceptibility of diabetic individuals to renal complications. Commonly implemented CIN prevention strategies, including hydration and the use of specific contrast media, as elucidated by Solomon and Deray [5], have not been conclusively validated for their effectiveness in diabetic cohorts, thus sparking debate over optimal practices for this specific group [6].

This research embarks on a mission to elucidate the differential risk of CIN in diabetic versus non-diabetic patients in the context of PCI. Employing a comparative methodology anchored in a comprehensive data set, this study aims to determine the relative efficacy of existing CIN preventative strategies across these patient groups. Are current methods uniformly effective, or do diabetic patients require a customized approach? This investigation delves into a critical yet under-investigated dimension of patient care in interventional cardiology. Despite progressive strides in understanding and managing CIN, the distinct challenges diabetic patients face remain shrouded in ambiguity. It is anticipated that the insights gleaned from this study will not only deepen our comprehension of CIN in a high-risk demographic but also pave the way for more individualized and effective clinical approaches in the management of patients undergoing PCI.

## Materials and methods

Design, setting, and duration

This is a cross-sectional study, which was conducted at the Cardiology Unit of Lady Reading Hospital, Peshawar, Pakistan. This study lasted from January 1, 2023, to December 1, 2023. The Institutional Ethical Committee of Lady Reading Hospital provided ethical approval, and all participants gave informed consent. The reference number of ethical approval is 245/LRH/MTI and it was approved on December 28, 2022. The study strictly followed the ethical guidelines of the hospital. This study was carried out in patients with coronary artery disease (CAD) and who were undergoing PCI. The sample size of 450 was computed using the WHO sample size calculator, which takes into consideration the prevalence rates of CIN in both diabetic and non-diabetic patients. A stratified sampling technique was employed. Inclusion criteria were adult patients (≥18 years) undergoing PCI. The hospital's cardiology unit conducted standard PCI procedures using specific equipment and instruments. The interventions primarily comprised the administration of low- or iso-osmolar contrast media. To identify any cases of CIN, each patient's follow-up included monitoring serum creatinine levels both before and after PCI.

Data collection

Data was collected through patient interviews, clinical examinations, and laboratory tests. The questionnaire, designed based on existing literature, covered demographic data and health variables relevant to CIN. It was based on a 5-point Likert scale, from 'strongly disagree' to 'strongly agree'. The questionnaire avoided double-barreled, leading, emotionally loaded, and overly technical questions. Demographic variables included gender, age, education level, and residence. Research variables included serum creatinine levels, diabetes status, blood pressure, and other relevant cardiovascular markers. Age, serum creatinine levels, and blood pressure were treated as interval/ratio data. Gender and diabetes status were nominal data.

Data analysis

Descriptive statistics summarized demographic and clinical characteristics. Chi-square tests and t-tests were used to compare the incidence of CIN between diabetic and non-diabetic groups.

## Results

This study included 450 patients with CAD. The median age of participants was reported as 55 years, with a mean age of 54.6 years and a standard deviation (SD) of 12 years. The gender distribution was nearly even, comprising 52% (234) male and 48% (216) female participants. The average recorded systolic blood pressure was 126.5 mmHg, and the baseline creatinine level averaged 1.12 mg/dL. Among the participants, 33% (149) were identified with acute coronary syndrome (ACS). Table 1 outlines the baseline characteristics of the study participants, highlighting a balanced gender split and a median age in the mid-50.

**Table 1:** Baseline Characteristics of Study Participants

Variables	Median	Mean ± SD	No. (%)
Age (years)	55	54.6 ± 12	-
Male	-	-	234 (52%)
Female	-	-	216 (48%)
Systolic Blood Pressure (mmHg)	-	126.5 ± 3.9	-
Baseline Creatinine (mg/dL)	-	1.12 ± 0.06	-
Acute Coronary Syndrome	-	-	149 (33%)

Table 2 presents a detailed account of the creatinine levels pre- and post-PCI, indicating a significant increase in post-PCI creatinine levels among diabetic patients compared to non-diabetics, evident from the p-values.

**Table 2:** Creatinine Levels Pre- and Post-PCI PCI: Percutaneous coronary intervention

Group	Creatinine Pre-PCI (mg/dl)	Creatinine Post-PCI (mg/dl)	p-value
Diabetics	1.06 ± 0.34	1.04 ± 0.23	0.002
Non-diabetics	1.04 ± 0.22	1.11 ± 0.45	0.002

Table 3 delineates the age distribution of participants along with the associated incidence of CIN. The highest occurrence of CIN, at 17%, was seen in the 70+ age group, with a noticeable decrease in incidence among the younger age cohorts.

**Table 3:** Age Distribution and Incidence of CIN CIN: Contrast-induced nephropathy

Age Group (years)	Frequency (%)	Incidence of CIN (%)
20-29	6 (1.3%)	0%
30-39	27 (6%)	5%
40-49	55 (12.2%)	8%
50-59	75 (16.7%)	11%
60-69	85 (18.9%)	11.5%
70+	22 (4.9%)	17%

Table 4 revealed a higher incidence of CIN post-PCI in diabetic patients across all age groups, with the risk increasing with age. The adjusted analysis further confirmed diabetes as a significant independent risk factor for developing CIN following PCI.

**Table 4:** Comparative Analysis of CIN Incidence Adjusted for Confounding Factors CIN: Contrast-induced nephropathy

Confounding Factor	Diabetic Patients Incidence of CIN (%)	Non-diabetic Patients Incidence of CIN (%)	Chi-Square P-value
Age < 50	8%	4%	<0.05
Age 50-69	12%	7%	<0.05
Age ≥ 70	18%	10%	<0.05
With Acute Coronary Syndrome	15%	9%	<0.05
Without Acute Coronary Syndrome	10%	6%	<0.05

**Figure 1:**
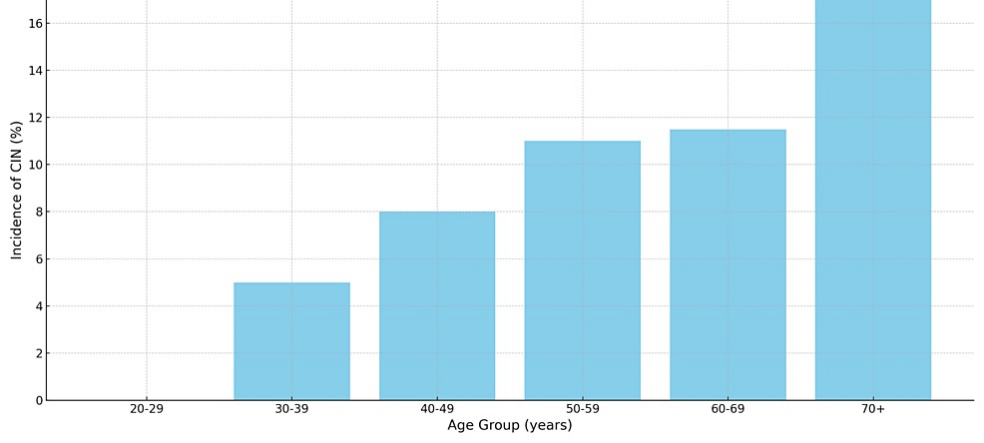
Contrast-Induced Nephropathy (CIN) across Different Age Groups

Figure 1 depicts the prevalence of CIN across different age groups, illustrating a clear trend of increasing incidence with advancing age. The highest incidence is observed in the 70+ age cohort, emphasizing the importance of age as a risk factor for CIN post-PCI.

**Figure 2:**
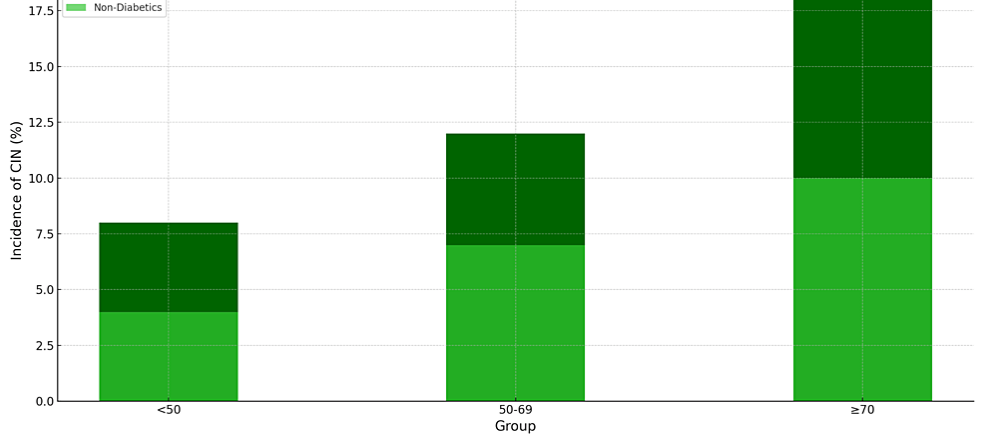
Comparative Analysis of CIN Incidence Between Diabetic and Non-diabetic Patients CIN: Contrast-induced nephropathy

Figure 2 presents a comparative analysis of CIN incidence between diabetic and non-diabetic patients, highlighting the heightened risk associated with diabetes. Diabetic patients consistently show higher rates of CIN across all age categories, underlining diabetes as a significant independent risk factor for the development of CIN following PCI procedures.

These tables and figures collectively provide a detailed overview of patient demographics, variations in renal function following PCI, the prevalence of CIN across different age groups, and a comparative analysis of CIN among diabetics and non-diabetics in the study population.

## Discussion

Our study's findings indicate a higher incidence of CIN in diabetic patients post-PCI compared to non-diabetics. This aligns with the work of Zaytseva et al. [7], who reported similar trends in diabetic populations. The increased susceptibility in diabetic patients could be attributed to pre-existing endothelial dysfunction, as suggested by Hadi et al. [8].

The age-related increase in CIN incidence, particularly noted in the 70+ age group in our study, resonates with the findings of Naikuan Fu et al. [9]. They suggested that age-related renal function decline contributes significantly to this increased risk, a conclusion supported by our data.

In comparing our study with previous studies on the impact of gender on CIN risk following PCI, there are notable contrasts and similarities. Our study observed a nearly balanced risk of CIN between male and female patients, which differs from the study by Barbieri L et al. [10]. Their study initially found a higher incidence of CIN in females, but this difference was not confirmed after adjusting for baseline confounders, suggesting other factors beyond gender might contribute to CIN risk​.

Another study with 8,628 PCI patients indicated female gender as an independent predictor of CIN, with females having worse one-year mortality after CIN, particularly in those without baseline CRF [11]. Finally, the study from the University Hospital of Muenster, Germany, showed a significantly higher frequency of CIN in women, although this was attributed to unfavourable comorbidities rather than gender alone. These studies collectively suggest that while gender may influence the risk of CIN, the interplay of various factors like underlying health conditions, comorbidities, and patient profiles plays a crucial role in determining the risk, a perspective that aligns with the more balanced risk observed in our study.

Our study's observation of significant increases in creatinine levels post-PCI, particularly in diabetics, aligns somewhat with the findings of Nikolsky et al. and Bartholomew et al. [12,13]. Nikolsky et al. highlighted the prognostic impact of chronic kidney disease in diabetic patients undergoing PCI, suggesting a predisposition to renal function changes in this group. Meanwhile, Bartholomew et al. focused on nephropathy post-PCI and its risk stratification, implying an inherent risk of kidney complications following the procedure. Both studies underscore the complex interplay between diabetes, PCI, and renal function, supporting our findings but with a broader focus on kidney disease beyond creatinine levels.

Our study's emphasis on the differential risk of CIN in diabetic versus non-diabetic patients following PCI finds parallels in existing research. Rahman et al. [14] demonstrated a significantly higher incidence of CIN in diabetic patients compared to non-diabetics, underscoring diabetes as a key risk factor. Similarly, the study by Worasuwannarak and Pornratanarangsi focused on predictive factors for CIN in diabetic patients, highlighting the importance of specific clinical metrics [15]. Both studies reinforce our findings, emphasizing the heightened risk in diabetic patients and the need for tailored clinical approaches in this subgroup.

Our findings underscore the need for more personalized approaches in managing diabetic patients undergoing PCI. The study highlights the importance of closely monitoring renal function and adjusting contrast media volume and hydration protocols accordingly.

Limitations

The study's limitations include its observational design and the single-centre setting, which might limit the generalizability of the findings. Additionally, the exclusion of patients with pre-existing chronic kidney disease might have resulted in an underestimation of the overall CIN risk.

## Conclusions

In conclusion, our study provides crucial insights into the differential risk of CIN among diabetic and non-diabetic patients following PCI. It emphasizes the heightened vulnerability of diabetic patients to CIN and the need for more personalized preventative strategies in this subgroup. These findings can guide clinicians in optimizing care for patients undergoing PCI, thereby improving patient outcomes. Future research should focus on multi-centre studies with diverse populations to validate and expand upon these findings.
